# Engineering vasculature: Architectural effects on microcapillary-like structure self-assembly

**DOI:** 10.1371/journal.pone.0210390

**Published:** 2019-01-08

**Authors:** Maria Isabella Gariboldi, Richard Butler, Serena M. Best, Ruth E. Cameron

**Affiliations:** 1 Cambridge Centre for Medical Materials, Department of Materials Science and Metallurgy, University of Cambridge, Cambridge, United Kingdom; 2 Imaging Facility, Gurdon Institute, University of Cambridge, Cambridge, United Kingdom; University of Texas at San Antonio, UNITED STATES

## Abstract

One of the greatest obstacles to clinical translation of bone tissue engineering is the inability to effectively and efficiently vascularize scaffolds. The goal of this work was to explore systematically whether architecture, at a scale of hundreds of microns, can be used to direct the growth of microcapillary-like structures into the core of scaffolds. Biphasic bioceramic patterned architectures were produced using silicone molds of 3D printed parts. Grooves and ridges were designed to have widths of 330 μm and 660 μm, with periodicities respectively of 1240 μm and 630 μm. Groove depth was varied between 150 μm and 585 μm. Co-cultures of human dermal microvascular endothelial cells (HDMECs) and human osteoblasts (hOBs) were used to grow microcapillary-like structures on substrates. Bioceramic architecture was found to significantly affect microcapillary-like structure location and orientation. Microcapillary-like structures were found to form predominantly in grooves or between convexities. For all patterned samples, the CD31 (endothelial cell marker) signal was at least 2.5 times higher along grooves versus perpendicular to grooves. In addition, the average signal was at least two times higher within grooves than outside grooves for all samples. Grooves with a width of 330 μm and a depth of 300 μm resulted in the formation of individual, highly aligned microcapillary-like structures with lengths around 5 mm. Extensive literature has focused on the role of nano- and micro-topography (on the scale below tens of microns) on cellular response. However, the idea that architecture at a scale much larger than a cell could be used to modulate angiogenesis has not been systematically investigated. This work shows the crucial influence of architecture on microcapillary-like structure self-assembly at the scale of hundreds of microns. Elucidating the precise correspondence between architecture and microcapillary-like structure organization will ultimately allow the engineering of microvasculature by tuning local scaffold design to achieve desirable microvessel properties.

## Introduction

Architecture across a wide range of length-scales has a crucial role in modulating biological response to scaffolds by affecting properties such as degradation behavior [1], nutrient perfusion [2], cell invasion [3], cell attachment [4] and differentiation [5]. Studies have recently focused on the crucial role played by macro-architecture at the hundred micron scale in modulating biological response by use of controlled bioceramic geometries. Specifically, Rumpler et al. [6] have demonstrated tissue growth is curvature-driven by analyzing the growth of cell layers on hydroxyapatite plates containing pores with different geometries *in vitro*. Gamsjäger et al. [7] have extended this model to account for surface stress, showing that tissue growth is higher on concave surfaces than on convex ones using hydroxyapatite substrates. Studies have translated these findings into computational models that have been shown to accurately predict the growth of tissue on complex, three-dimensional geometries [8].

Currently, one of the greatest challenges faced by tissue engineering is the inability to efficiently vascularize tissue engineering constructs [9]. The failure to extensively vascularize biomaterials following implantation results in limited nutrient and waste exchange to cells within the implant, which can ultimately result in necrotic core formation and implant failure [10]. This challenge of vascularization has limited the size of defects that can currently be repaired using bioceramic scaffolds, restricting the prospect of bone tissue engineering as a means to replace large bone defects. Several approaches have been taken to tackle this issue, such as embedding or functionalizing scaffolds with pro-angiogenic factors such as vascular endothelial growth factor (VEGF) [11] or basic fibroblast growth factor (bFGF) [12].

Co-cultures of endothelial cells and osteoblasts, or osteoblast-like cells, have been shown to result in the self-assembly of endothelial cells into microvessel-like tubular structures *in vitro*, which could act as functional vasculature, as demonstrated by the presence of a lumen [13]. These structures have been shown to successfully anastomose with host vasculature following implantation [14]. This method of *in vitro* vascularization has been proposed to be both a useful model for *in vivo* vascularization and a clinically useful tool for pre-vascularizing biomaterial constructs prior to implantation using patient-derived cells [15]. Multiple studies have mentioned the effect of architecture on angiogenesis or neovascularization *in vivo* [16, 17]. However, none of these studies have compared architectures in a systematic way by independently varying individual architectural parameters. Further, none of these studies involve self-assembly of microcapillary-like structures using co-cultures, a distinct process from sprouting angiogenesis *in vivo* with promising prospects for clinical use.

The purpose of this study is to explore the effect of macro-architecture on microcapillary-like structure formation and organization on architecturally controlled biphasic (hydroxyapatite/α-tricalcium phosphate) bioceramic substrates using co-cultures. Modulating the formation of self-assembled microcapillary-like structures using macro-architecture would benefit the production of pre-vascularized biomaterials for clinical use. We show that concavities allow the patterning of angiogenesis by controlling the position and direction of microcapillary-like structures on bioceramic surfaces.

## Materials and methods

### Fabrication of negative molds using curable silicone

**Fig 1:**
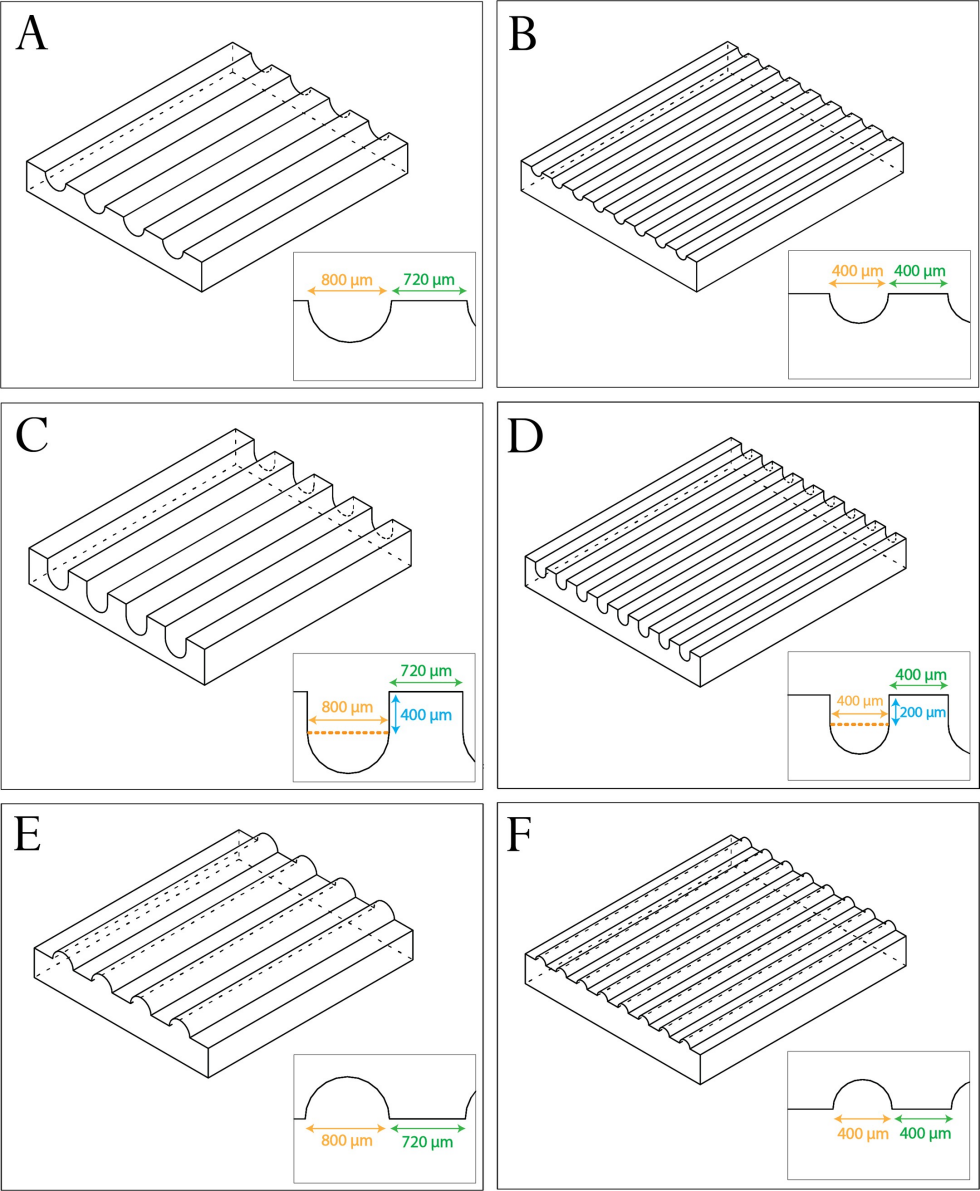
Designs of 3D printed architectures used to explore the effects of concavities and convexities on microcapillary-like structure self-assembly. Designs can be divided into large radii of curvature of 400 μm (A, C and E) and small radii of curvature of 200 μm (B, D and F). For each radius of curvature there is a standard concavity sample (A and B), a deep concavity sample (C and D) and a convex equivalent sample (E and F). Deep concavities had a cross section in which the semi-circular concavity had an additional depth equivalent to the radius of curvature.

Desired architectures were designed using freely available computer aided design (CAD) software SketchUp (Trimble Navigation, USA). An overview of the chosen architectures can be seen in Fig 1. Architectural designs were inspired by previous studies on biological response to architecture [6, 7, 18]. The combination of architectures was selected to allow systematically testing the effect of concave versus convex features as well as the effects of radius of curvature and depth of concavities. Flat samples were used as controls. All surfaces were designed on 6.8 mm x 8 mm rectangles. Parts were 3D printed and mail delivered by Shapeways (NL) in the material Frosted Extreme Detail Plastic. This material was printed at a very high resolution using Multi-Jet Modeling (MJM) process, allowing for a 16 μm layer height. Negative molds of the resulting plastic architectures were cast using Polycraft GP-3481-F Silicone Rubber (MB Fibreglass, UK) using a 1:10 ratio of curing agent to liquid silicone, as indicated by the manufacturer's instructions, and cured overnight.

### Bioceramic surface fabrication

Hydroxyapatite powder with a 10 μm particle size was purchased from Sigma Aldrich (UK). The original hydroxyapatite powder used was phase-pure but was found to decompose into α-TCP upon heat treatment. The powder was calcined at 900°C for one hour with a heating rate of 5°C /min and a cooling rate of 4°C /min. Calcination was performed in humidified argon atmosphere.

The particle size distribution of the suspension was analyzed using a MasterSizer (Malvern Instruments Ltd., UK). A slurry was made by adding gelatin from bovine skin (Sigma Aldrich, UK) to a 50 weight% hydroxyapatite suspension in water. The gelatin was added as a binder to the hydroxyapatite suspension at a concentration of 0.03 g/mL and dissolved by briefly heating the slurry at 70°C and carefully mixing. The resulting slurry was used to cast the silicone molds, dried overnight and sintered at 1200°C for one hour with a heating rate of 5°C /min and a cooling rate of 4°C /min in humidified argon atmosphere.

### Characterization of hydroxyapatite powders and surface architectures

For scanning electron microscopy (SEM) imaging, hydroxyapatite powders and sintered samples were gold-coated and imaged using a 5800LV scanning electron microscope (JEOL, USA) at a 10 kV operating voltage. X-ray diffraction (XRD) was performed on calcined powders and final sintered samples. XRD was performed using a Philips PW1820 Theta/2-Theta diffractometer with Bragg-Brentano para-focusing geometry.

### Co-culture experiments

Human dermal microvascular endothelial cells (HDMEC) and human osteoblasts (hOBs) were purchased from PromoCell GmbH (Germany). HDMECs were cultured in Endothelial Basal Medium MV (PromoCell) supplemented with 15% FBS (Sigma Aldrich, UK), 10 μg/mL heparin sodium salt from porcine mucosa (Sigma Aldrich, UK), 2.5 ng/mL human recombinant bFGF (Gibco, UK) and penicillin/streptomycin (Sigma Aldrich, UK) according to the protocol of Unger et al. [13]. hOBs were cultured in complete Osteoblast Growth Medium (PromoCell GmbH, Germany). Cells were passaged at high confluence (> 90%) and split to low ratios (generally 1:3) to avoid loss of phenotype.

All samples were used in triplicate and all co-cultures were grown in HDMEC media. Samples were sterilized by heating at 200°C, placed in low adhesion 24-well plates and washed once with PBS (Gibco, UK). Samples were pre-incubated in endothelial media before seeding to avoid cell localization being affected by capillarity. 71,250 of each HDMECs and hOBs at passage 6 were seeded in each well and allowed to attach overnight. Total cell densities were chosen based on the co-culture protocol by Unger et al. (2007). A 1:1 cell ratio was established to be optimal with the specific cells being used in previous optimization experiments. Higher passage numbers were avoided to prevent phenotype loss. Samples were subsequently moved to fresh wells after which media was changed every 2–3 days.

### Immunofluorescence and imaging

At day 7, samples were washed once with PBS and fixed using 4% paraformaldehyde solution in PBS (Affymetrix, UK) for 10 minutes at room temperature. Samples were washed three times with PBS. Samples were blocked in 1% BSA (Sigma Aldrich, UK) for one hour and stained using anti-human CD31 pre-conjugated Alexa 488 antibody (BioLegend, UK) at a 1:100 dilution for one hour and DAPI (Sigma Aldrich, UK) at a 1:5000 dilution. Samples were washed with PBS and immobilized on 24 well plate wells using Vectashield antifade mounting medium (Vector Laboratories Ltd, UK).

All imaging was performed using an Opera Phenix^TM^ High-Content Screening System (Perkin-Elmer, USA) using a 5x objective. This imaging system allows for high throughput imaging of entire 24-well plates.

### Image analysis of directionality

Image stacks were reconstructed using Wagner (https://github.com/gurdon-institute/Wagner/releases/tag/v1.0). Whole sample fluorescent image stacks were analyzed to quantify microcapillary-like structure alignment using the ImageJ Directionality plugin.

Stacks were rotated so that the groove direction corresponded to 0°, and cropped to a size of 5740 μm x 5023 μm (2400 x 2100 pixels) to exclude sample edges. The background signal was subtracted from maximum intensity projections using a rolling ball radius of 50 pixels. For alignment quantification, Directionality plugin (Tinevez, release 2.0.2) results using Fourier component analysis were compared with groove orientation. The degree of alignment (DOA) was defined as the ratio of the signal along the grooves (0°) to the signal perpendicular to the grooves (90°).

### Image analysis of endothelial structure localization

Rotated stacks were cropped to a size of 5740 μm x 5023 μm (2400 x 2100 pixels) to exclude sample edges and the background signal was subtracted from maximum intensity projections using a rolling ball radius of 50 pixels. The image thresholds were adjusted by auto-contrasting before converting them to 8-bit grayscale. Phansalkar thresholding was applied using a radius of 5 pixels. Finally, all particles with sizes below 400 μm^2^ were removed. Images rotated to have vertical grooves were analyzed to produce vertically averaged pixel intensity data across the sample length.

In order to detect grooves, we developed the image plugin GrooveJ (https://github.com/gurdon-institute/GrooveJ/releases/tag/v1.1.0). A line width for depth profiles of 300 μm, a curvature of 0.4, a rolling Z window of 100 μm, a Z threshold of 0.5, a slope factor of 0.35, and a depth weighting of 0.01 were used. Lines for groove detection were drawn across the sample in the central region of the cropped sample stacks. Groove detection data and vertically averaged pixel intensity data were imported and analysed using Matlab (MathWorks, Inc., Natick, MA, United States). The degree of containment (DOC) was defined as the ratio of the average signal intensity in grooves (total signal in grooves normalized to the total cross-sectional length of grooves) to the average signal intensity outside of grooves (total signal outside of grooves normalized to the total cross-sectional length of regions outside grooves). For convex ridges, grooves were considered as the regions between convexities.

## Results

### Characterization of hydroxyapatite powders and surface architectures

**Fig 2:**
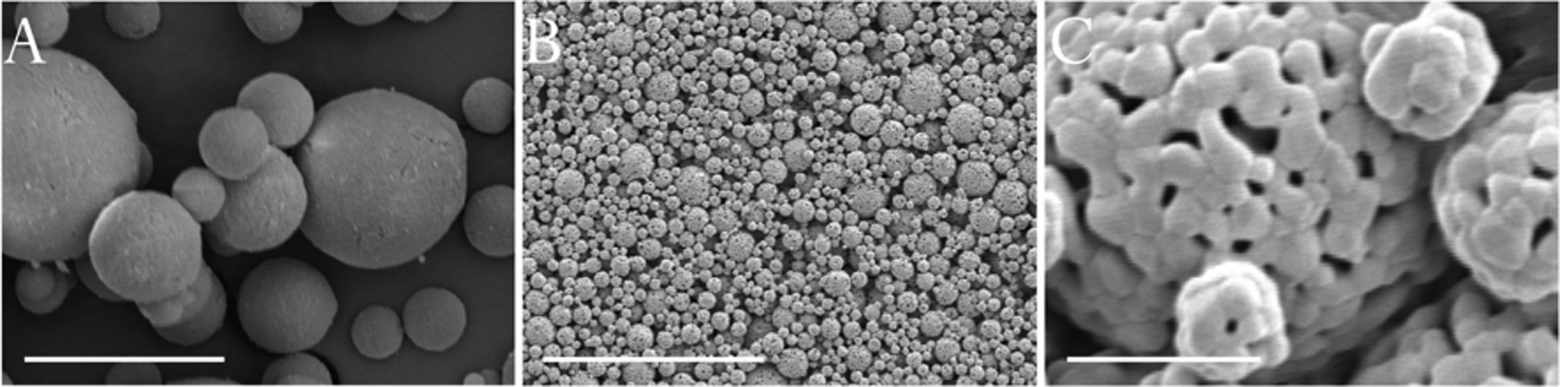
SEM micrographs of ceramic materials. Images show original hydroxyapatite powder particles (A) and final sintered structures (B and C). Scalebars are (A) 10 μm, (B) 50 μm and (C) 3 μm.

Particle sizing of calcined powders showed a particle size distribution with a d(0.1) of 3.77 μm, a d(0.5) of 10.91 μm and a d(0.9) of 33.04 μm. While the original powder was found to be phase-pure hydroxyapatite according to XRD analysis, it progressively decomposed into α-TCP upon heat treatment. The ratio of the highest α-TCP peak and the highest hydroxyapatite peak, respectively at 2θ values of 30.75° and 31.77°, were used as an estimation of the relative weight ratio of the two phases [19]. Calcined hydroxyapatite powder was found to have approximately 2.0% α-TCP content, whereas final sintered structures were found to have approximately 20.3% α-TCP. Powder particles had smooth spherical surfaces (Fig 2A), which packed to produce a homogenous surface topography (Fig 2B). The original powder morphology is visible in the final sintered structures (Fig 2C).

**Fig 3:**
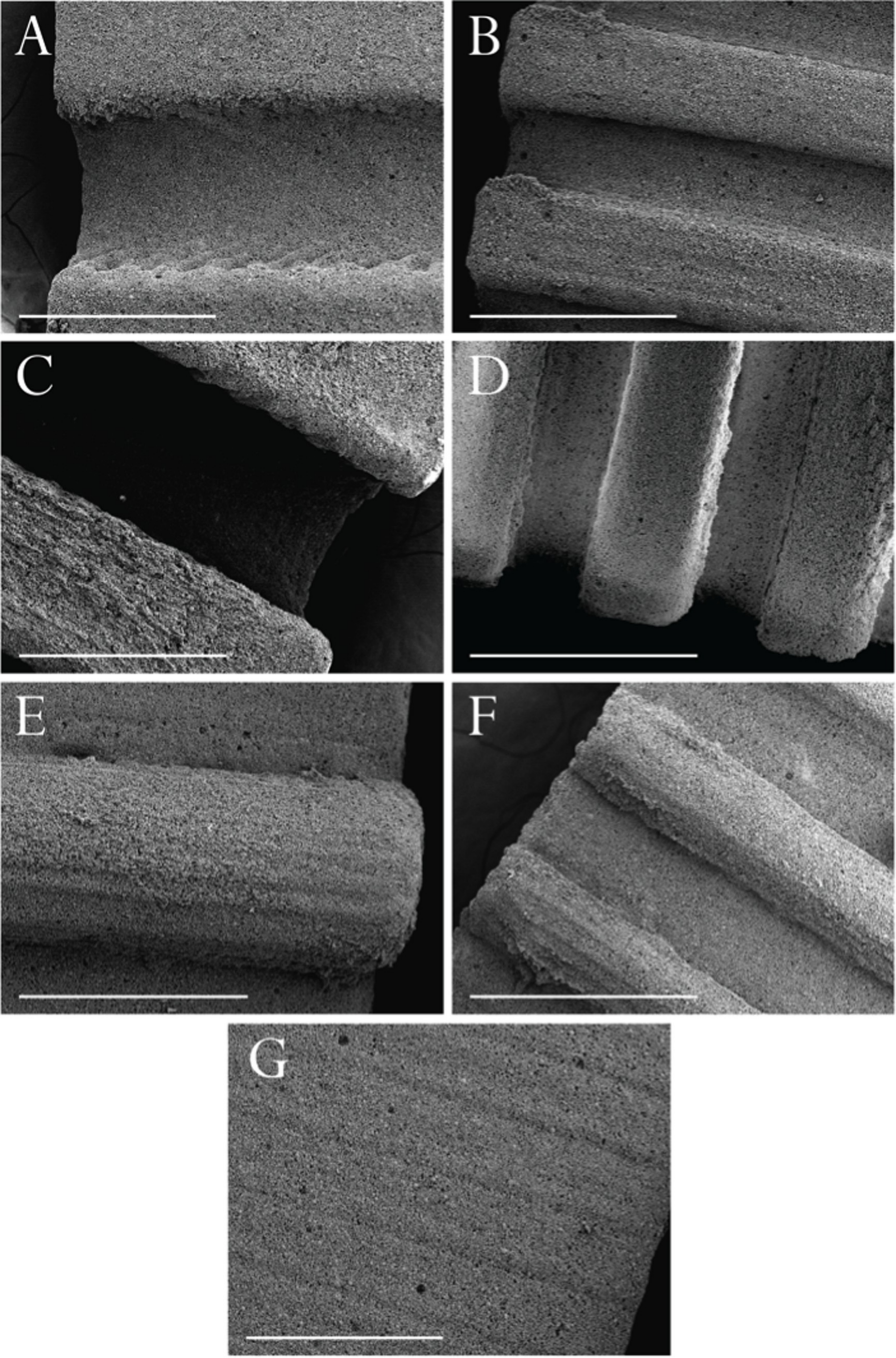
SEM micrographs of sintered bioceramic structures. Images show (A) large concavities, (B) small concavities, (C) large deep concavities, (D) small deep concavities, (E) large convexities, (F) small convexities and (G) flat controls. Scalebars are 600 μm.

Fig 3 shows final biphasic bioceramic structures. The use of liquid silicone for the production of molds allowed the fabrication of high-resolution architectures with high fidelity to original designs. Shrinking resulted in large features (Fig 3A, 3C and 3E) having a diameter of 660 μm and a periodicity of 1240 μm. Large concavities (Fig 3A) and large convexities (Fig 3E) had a depth/height of 300 μm and large deep concavities (Fig 3C) had a depth of 585 μm. Small features (Fig 3B, 3D and 3F) had a diameter of 330 μm and a periodicity of 630 μm. Small concavities (Fig 3B) and small convexities (Fig 3F) had a depth/height of 150 μm and small deep concavities (Fig 3D) had a depth of 300 μm. The MJM method used for original plastic parts resulted in a characteristic surface micro-structure due to liquid droplet deposition observable in ceramic replicates. This characteristic was particularly visible in flat samples when imaged using SEM (Fig 3G).

### Architectural effects on microcapillary-like structure self-assembly

**Fig 4:**
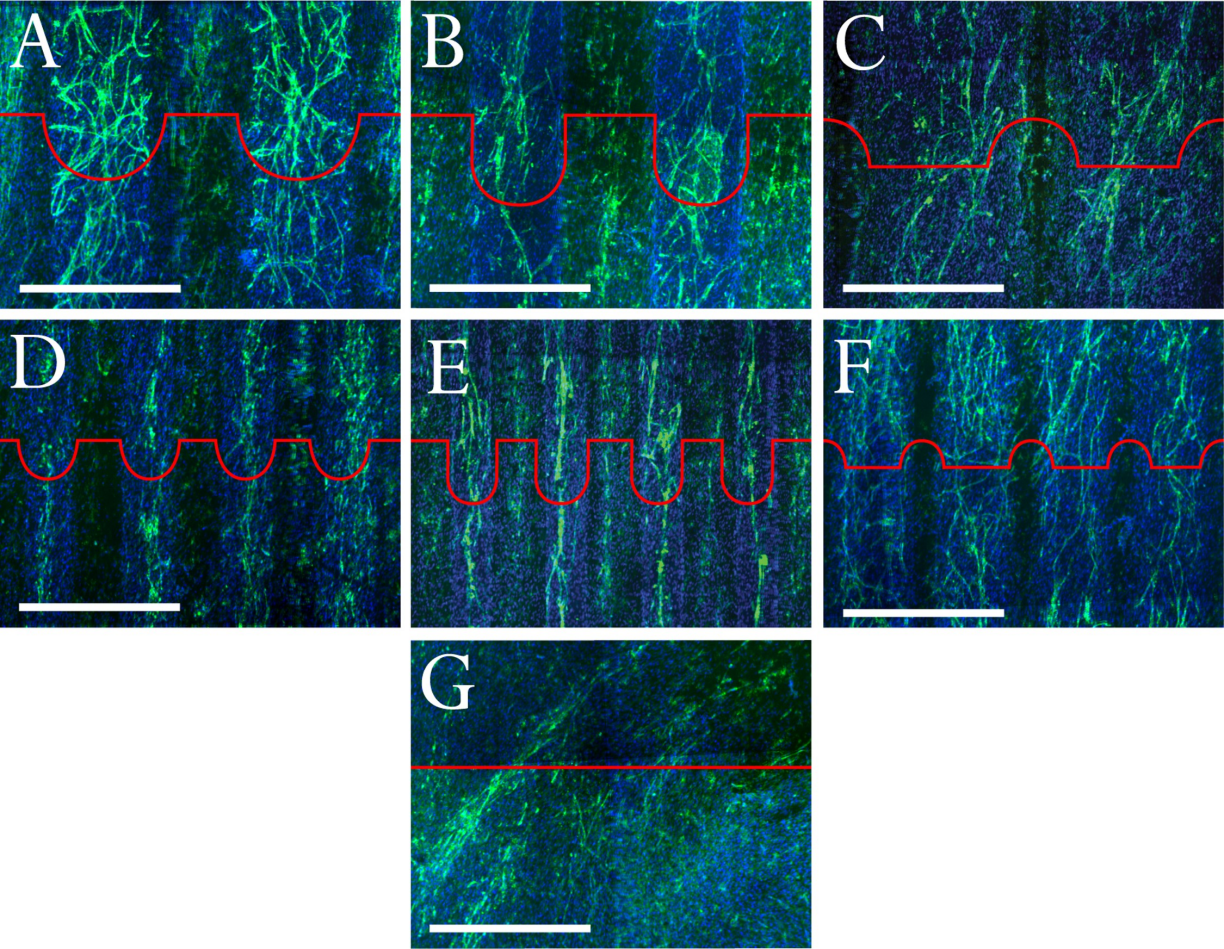
Confocal images of co-cultures stained with DAPI and immunofluorescently stained for CD31 to show self-assembled microcapillary-like structures. Images display microcapillary-like structures on (A) large concavities, (B) large deep concavities, (C) large convexities, (D) small concavities, (E) small deep concavities, (F) small convexities and (G) flat controls. Red profiles illustrate substrate architecture. Scalebars are 500 μm.

Macro-architecture significantly influenced the location, shape and alignment of self-assembled microcapillary-like structures (Fig 4). For concave samples (Fig 4A, 4B, 4D and 4E), microcapillary-like structures formed predominantly in concavities. In large concavities (Fig 4A), the microcapillary-like structures formed highly branched networks predominantly located inside grooves. Microcapillary-like structures were also found to form in flat areas of these samples, with a higher alignment with groove direction. Small grooves (Fig 4D) also resulted in endothelial cell structures predominantly located within the grooves. However, these structures were less defined and were present in smaller areas of the samples. Wider concavities (Fig 4A and 4B) were found to produce microcapillary-like structures with more lateral branching than narrower concavities (Fig 4D and 4E).

Deep concavities resulted in more contained microcapillary-like structures (Fig 4B and 4E) compared with shallower concavities (Fig 4A and 4D). Large deep concavities often resulted in the formation of endothelial cell structures larger than individual microcapillary-like structures (Fig 4B). Small deep concavities resulted in individual, highly aligned, microcapillary-like structures almost exclusively inside concavities (Fig 4E).

For both large and small convexities (Fig 4C and 4F), microcapillary-like structures formed between convexities. The tops of concavities almost completely inhibited microcapillary-like structure network continuity. Flat control samples displayed fewer, disorganized microcapillary-like structures (Fig 4G). Whole sample images can be found in Supplementary Material (S1 Fig). The striated micro-structure resulting from the MJM process particularly evident in flat samples (Fig 3G) was not found to result in any observable effects on cell behavior.

### Image analysis for CD31 signal quantification

**Fig 5:**
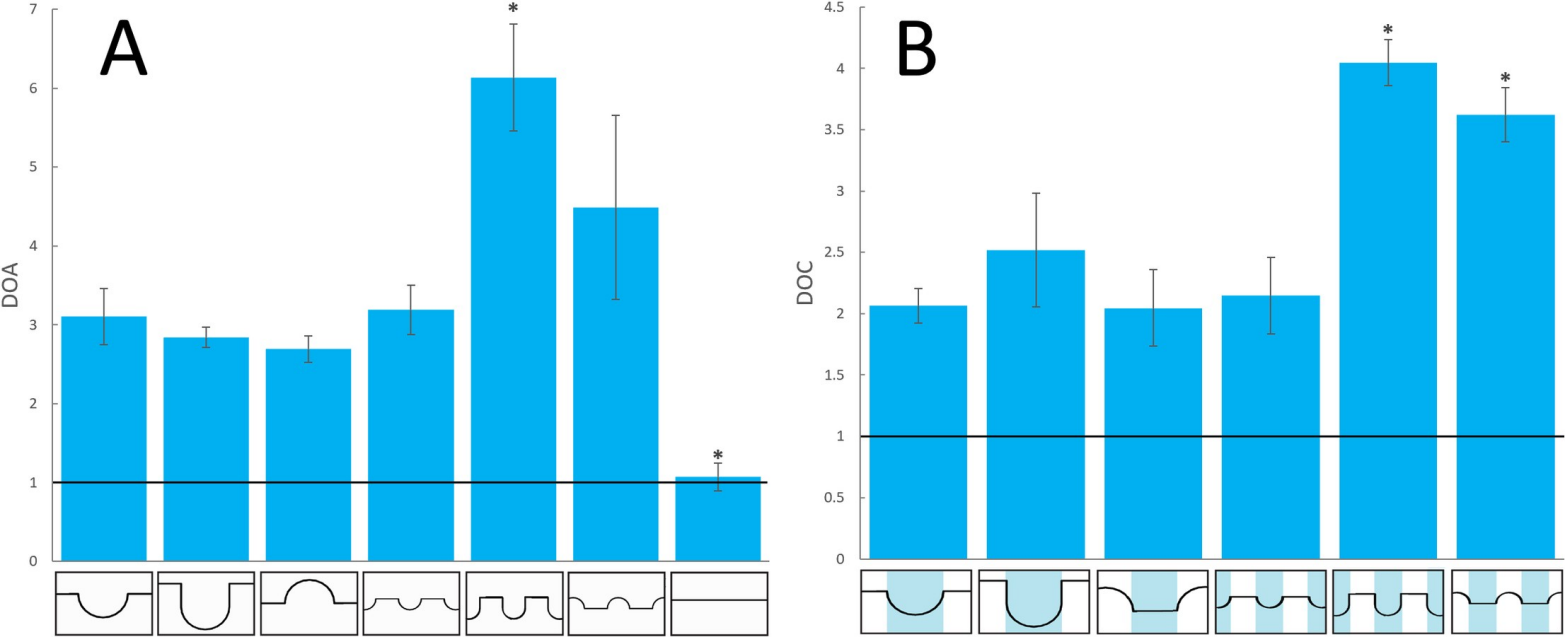
DOA and DOC signal analysis measurements. Mean (A) DOA and (B) DOC measurements of CD31 signal of co-cultures grown on bioceramic substrates with different architectures (n = 3 for each). Error bars indicate standard errors of the means. Measurements were performed on a 5740 μm x 5023 μm area of the maximum intensity projection of each triplicate. Shading in the DOC structure graphical representations highlights areas that were considered as grooves for signal containment analysis. Lines at y = 1 indicate random signal alignment for DOA and uniform microcapillary-like structure distribution for DOC. * indicates values found to be significantly different from other architectures (Kruskal-Wallace with Dunn's post-hoc test, P<0.05). Mean DOC values for small deep and small convex architectures are not significantly different from each other.

The results from the degree of alignment (DOA) and the degree of containment (DOC) analysis are shown in Fig 5A and 5B respectively. Flat samples resulted in an average DOA close to 1, as would be expected from random signal alignment. All patterned architectures were found to align microcapillary-like structures, with the lowest DOA being 2.69 for large convexities. Low DOAs can be explained as more random vessel alignment or higher degrees of lateral branching. The Kruskal-Wallace test showed multimodal distributions for both DOA and DOC (P<0.05). Dunn's post-hoc test indicated that DOA values for all other architectures were significantly different from flat controls (P<0.005 in all cases), while values for large, large deep, large convex and small architectures were similar overall with only large convexities and small concavities showing a significant difference from each other (P<0.005).The highest DOA was exhibited by the small deep concavities, in line with imaging result showing the formation of individual, highly aligned microcapillary-like structures in these architectures. Dunn's test on the DOC values showed that small deep and small convex architectures had values larger than other architectures and similar to each other. The significance cutoff used in all cases was P<0.05.

Details of the image processing procedure, including image binarization and filtering procedure results, groove detection and signal analysis, can be found in the Supplementary Material. The image processing procedure resulted in low background images with no visible loss of signal (S2 and S3 Figs). Examples of the results of groove detection using GrooveJ and vertically averaged signal profiles for each architecture can be found respectively in S4 and S5 Figs. DOC measurements displayed similar trends to DOA measurements, with the highest containment displayed by small convexities and small deep concavities (Fig 5B). Large deep concavities displayed lower DOC scores than expected from the observation that most vessels were localized in grooves. This is likely to be the result of vessels growing on groove edges combined with an overall lower number of vessels, resulting in an underestimation of microcapillary-like structures due to a lower signal to noise ratio. This demonstrates some of the limitations in using a signal analysis approach.

## Discussion

We show for the first time the effects of systematically varying macro-architecture on microcapillary-like structure self-assembly using co-cultures by varying convexity and concavity radius of curvature and concavity depth. The methods developed can be viewed as a toolkit that can be extended to other 2.5D architectures and other castable biomaterials to further explore architectural effects on microcapillary-like structure self-assembly, leading towards a predictive model for architectural optimization for angiogenic control similar to models developed by Rumpler et al. [6] and Guyot et al. [8] for cell network growth on complex structures.

Our findings on the positive role of concavities are in line with *in vivo* studies performed by Scarano et al. [17], who found that vessels concentrate in concavities in threaded titanium implants in rabbit tibia models. The role of concavities has been previously discussed in the literature as an inductive geometric cue for bone tissue growth [18, 20]. These studies view concavities as biomimetic signals that mimic trenches left by osteoclasts during the bone resorption process and promote hemi-osteon formation. In the context of angiogenesis, grooves, and particularly small grooves, can be seen as biomimetic cues analogous to Haversian canals, which host vasculature in the bone structure.

Mechanistic explanations for observed architectural effects can be divided into biochemical and biomechanical hypotheses. Concavities and spaces between convexities can provide locally higher concentrations of pro-angiogenic factors by acting as physical barriers, as suggested by Chiu et al. [21] in their use of grooves to direct sprouting outgrowths from mouse vascular explants. Convexities and concavity edges could also be acting as biomechanical inhibitors by affecting cell network growth, as suggested by Bidan et al. [18] in their “chord model” representation of cell layers acting as tensile networks that illustrated the inhibitory effect of convexities on cell growth. Computational models would help further elucidate the degree to which biochemical and biomechanical effects contribute to the clear influence of architecture on microcapillary-like structure self-assembly. In the context of biomechanics, a useful starting model could be provided by the differential adhesion hypothesis (DAH). This hypothesis models tissues composed of different cell types as liquids with different surface tensions resulting from differences in intercellular adhesion molecules and has been shown to accurately predict cell morphogenetic and self-assembly processes such as cell segregation [22]. This approach could be used to model both cell-substrate interactions, as a wetting problem, and the segregation of endothelial cells from osteoblasts during self-assembly as the interaction between two fluids with different surface properties. This would allow the combination of the findings related to curvature-driven growth by Bidan et al. [18] with the findings of Foty and Steinberg [22] on cell aggregate surface tension effects on cell segregation and self-assembly.

Combining architectural control with cellular self-assembly allows the production of more extensive networks in shorter time-scales than achievable through sprouting angiogenesis. Chiu et al. [21] reported the directed growth of explant outgrowths over distances up to 700 μm in 14 days. Sprouting angiogenesis is a distinct process from microcapillary-like structure self-assembly used in this study, in that it relies on the gradual growth of a tubule from a vascular structure “source”. Here, the microcapillary-like structures produced using small deep concavities, for example, spanned approximately 5 millimeters after only 7 days (S1D Fig) with no exogenous angiogenic stimuli. Use of *in situ* cell self-assembly allows the production of more extensive microcapillary-like structures by overcoming the higher time-dependence of sprouting angiogenesis.

We showed that architecture can be used to control both the localization and orientation of self-assembled microcapillary-like structures. Architecture alone is sufficient to regulate microcapillary-like structure growth, thus allowing for a more elegant and biologically controlled way of directing vascularization into the core of a scaffold than methods such as biomolecule encapsulation. The very promising results here have been obtained by systematically comparing six architectures. This process can be viewed as a toolkit that can easily be extended to systematically screen more 2.5D architectures in a high throughput manner by changing the original 3D printed part design.

Further, this work has important implications for additive manufacturing techniques used for biomaterial production. Extrusion technology is commonly employed as a way of producing bioceramic and soft biomaterial constructs. This method inherently produces convex surfaces, which would not be favorable for microcapillary-like structure self-assembly. More importantly, this work has important clinical implications, with architecture allowing more efficient vascularization by directing vascular structures to the core of biomaterials, thus overcoming the issue of core degradation in implanted constructs.

## Conclusions

Six architectures consisting of concave grooves and convex ridges were used to systematically screen structural effects at a scale of hundreds of microns on microcapillary-like structure self-assembly by varying radius of curvature and depth. Architecture at a scale much larger than individual cells was found to significantly affect microcapillary-like structure self-assembly down to individual microcapillary-like structure control in small deep grooves. Microcapillary-like structure networks were found to form predominantly in concavities and between convexities, with convex structures inhibiting network continuity. Both ridges and grooves were found to affect both CD31 signal localization and directionality.

The work presented constitutes an accessible toolkit that can be employed for the production of high-resolution bioceramic architectures and the screening of architectures for their angiogenic potential. A more comprehensive screen of architectural effects on microcapillary-like structure self-assembly with systematic parameter variation will allow uncovering the precise mechanism for architectural effects. Results obtained showing architectural control of microcapillary-like structure self-assembly can be viewed both as a model for *in vivo* biomaterial vascularization and as a tool to better control *in vitro* pre-vascularization of constructs for later implantation. This could lead towards the ability to engineer vasculature by locally optimizing architecture for desirable microcapillary properties.

## Supporting information

S1 Fig Whole sample confocal images of co-cultures stained with DAPI and immunofluorescently stained for CD31. Images show self-assembled microcapillary–like structures on (A) large concavities, (B) small concavities, (C) large deep concavities, (D) small deep concavities, (E) large convexities, (F) small convexities and (G) flat controls. Scalebars are 1 mm.
(TIF)

S2 Fig Binarization procedure output examples. Demonstration of the developed binarization procedure’s ability to detect microcapillary-like structures in two distinct architectures: large concavities (top) and small deep concavities (bottom). Images are 2.58 mm x 2.58 mm.
(TIF)

S3 Fig Binarization procedure example. Example of the binarization pipeline applied to an individual tile illustrating the effect of each step on the vertically averaged signal profile. Overall signal is maintained with a lowering of baseline background to levels close to zero for images after particle removal, demonstrating no significant loss of information. Images are 2.58 mm x 2.58 mm.
(TIF)

S4 Fig Groove detection results by GrooveJ plugin used for signal localization measurements. Images show sample results for (A) large concavities, (B) small concavities, (C) large deep concavities, (D) small deep concavities, (E) large convexities and (F) small convexities.
(TIF)

S5 Fig Signal localization analysis used for DOC measurements. Vertically averaged signal results superimposed to detected grooves in pink for one sample of each (A) large concavities, (B) small concavities, (C) large deep concavities, (D) small deep concavities, (E) large convexities and (F) small convexities.
(TIF)
