# Supplementary figures and images for: Engineering vasculature: Architectural effects on microcapillary-like structure self-assembly
Source: PLoS One. 2019 Jan 8;14(1):e0210390. doi: 10.1371/journal.pone.0210390 (PMC6324810; doi:10.1371/journal.pone.0210390)

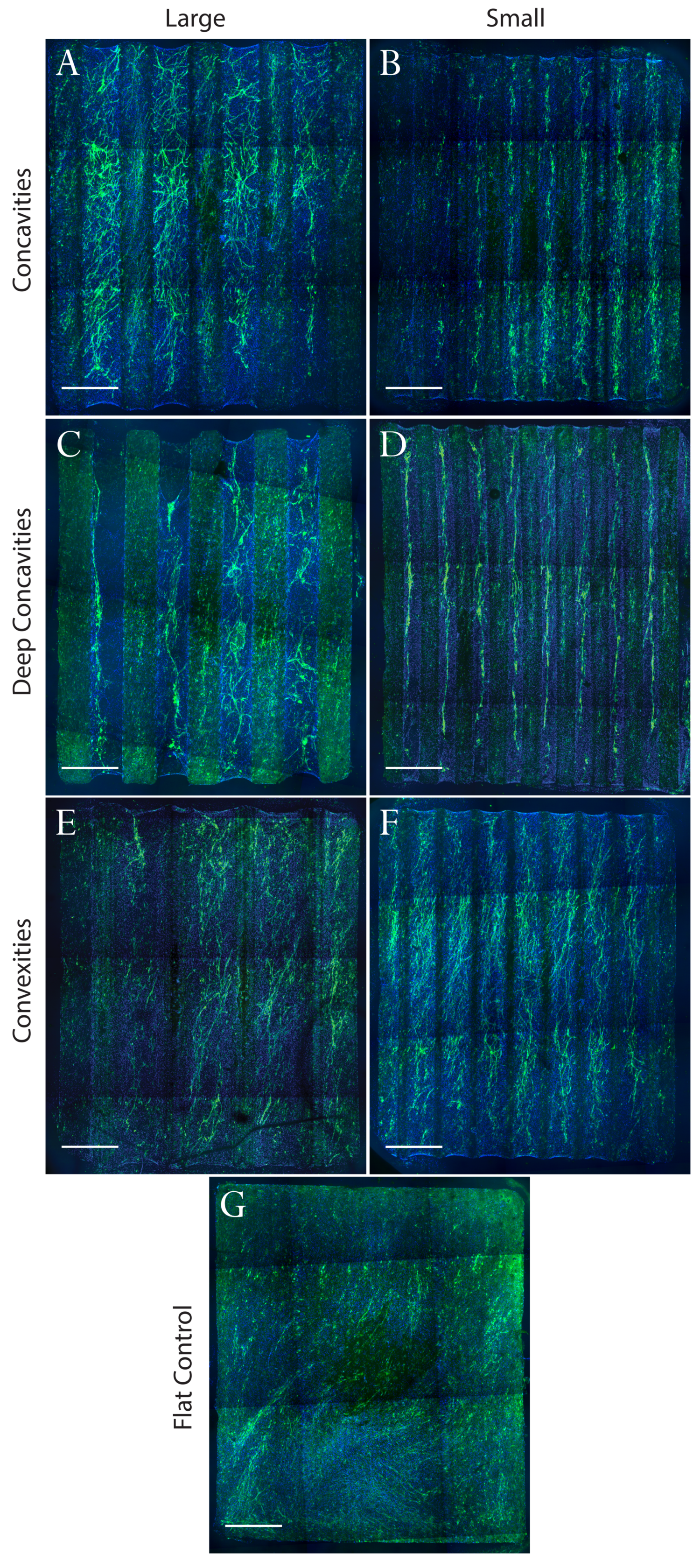

Supplement: S1 Fig — Images show self-assembled microcapillary–like structures on (A) large concavities, (B) small concavities, (C) large deep concavities, (D) small deep concavities, (E) large convexities, (F) small convexities and (G) flat controls. Scalebars are 1 mm. (TIF) [file pone.0210390.s001.tif]

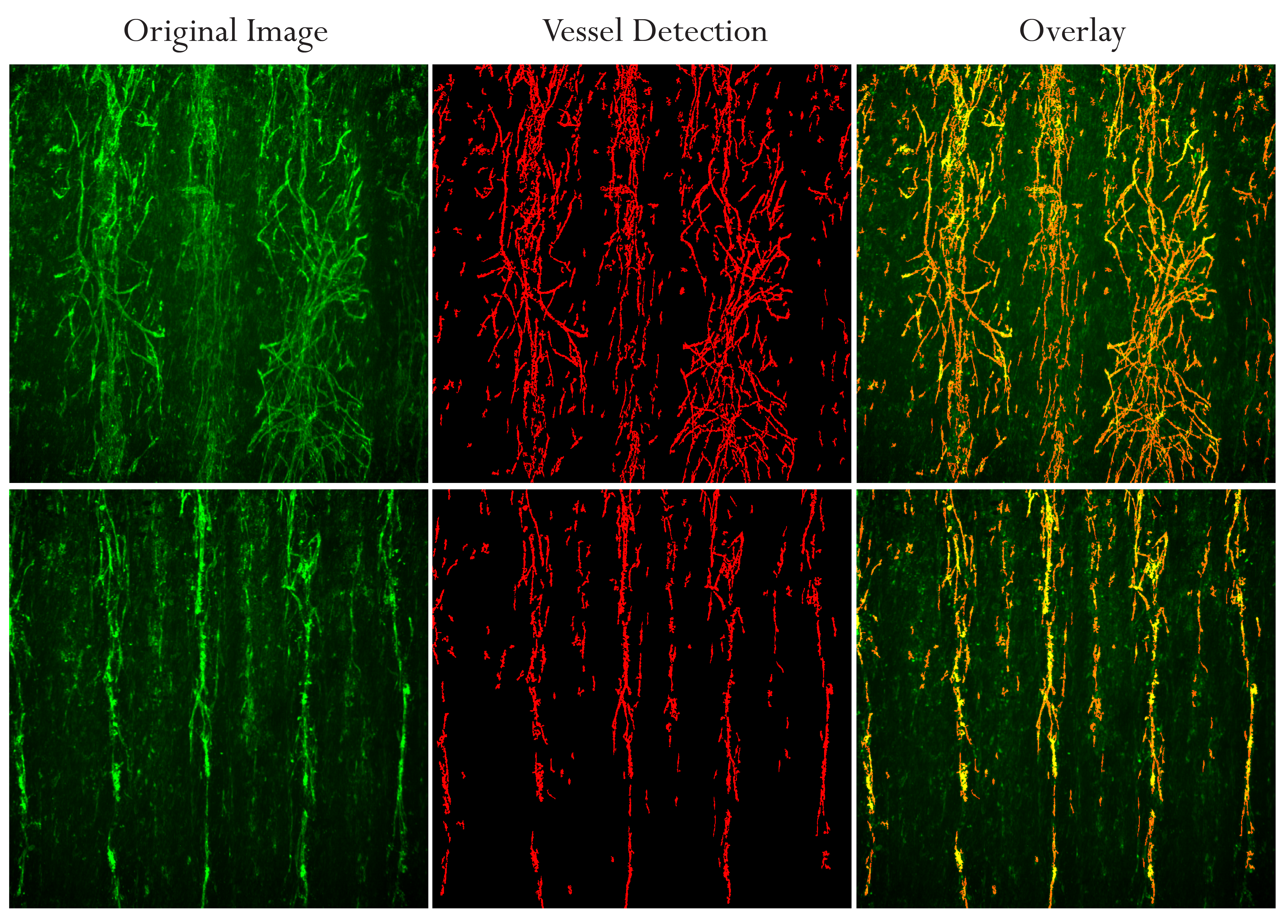

Supplement: S2 Fig — Demonstration of the developed binarization procedure’s ability to detect microcapillary-like structures in two distinct architectures: large concavities (top) and small deep concavities (bottom). Images are 2.58 mm x 2.58 mm. (TIF) [file pone.0210390.s002.tif]
